# Cutaneous C-polymodal fibers lacking TRPV1 are sensitized to heat following inflammation, but fail to drive heat hyperalgesia in the absence of TPV1 containing C-heat fibers

**DOI:** 10.1186/1744-8069-6-58

**Published:** 2010-09-21

**Authors:** H Richard Koerber, Sabrina L McIlwrath, Jeffrey J Lawson, Sacha A Malin, Collene E Anderson, Michael P Jankowski, Brian M Davis

**Affiliations:** 1 Department of Neurobiology, School of Medicine, University of Pittsburgh, 3500 Terrace St, Pittsburgh, PA 15261, USA; 2 Department of Medicine, University of Pittsburgh, 3550 Terrace St, Pittsburgh, PA 15261, USA

## Abstract

**Background:**

Previous studies have shown that the TRPV1 ion channel plays a critical role in the development of heat hyperalgesia after inflammation, as inflamed TRPV1-/- mice develop mechanical allodynia but fail to develop thermal hyperalgesia. In order to further investigate the role of TRPV1, we have used an ex vivo skin/nerve/DRG preparation to examine the effects of CFA-induced-inflammation on the response properties of TRPV1-positive and TRPV1-negative cutaneous nociceptors.

**Results:**

In wildtype mice we found that polymodal C-fibers (CPMs) lacking TRPV1 were sensitized to heat within a day after CFA injection. This sensitization included both a drop in average heat threshold and an increase in firing rate to a heat ramp applied to the skin. No changes were observed in the mechanical response properties of these cells. Conversely, TRPV1-positive mechanically insensitive, heat sensitive fibers (CHs) were not sensitized following inflammation. However, results suggested that some of these fibers may have gained mechanical sensitivity and that some previous silent fibers gained heat sensitivity. In mice lacking TRPV1, inflammation only decreased heat threshold of CPMs but did not sensitize their responses to the heat ramp. No CH-fibers could be identified in naïve nor inflamed TRPV1-/- mice.

**Conclusions:**

Results obtained here suggest that increased heat sensitivity in TRPV1-negative CPM fibers alone following inflammation is insufficient for the induction of heat hyperalgesia. On the other hand, TRPV1-positive CH fibers appear to play an essential role in this process that may include both afferent and efferent functions.

## Introduction

The transient receptor potential vanilloid type 1 (TRPV1) ion channel is activated by a variety of stimuli including capsaicin, heat, acidic pH and anandamide [1-4]. In addition, TRPV1 channel function can be modulated by numerous endogenous compounds released following injury, or inflammation including ATP, histamine, bradykinin, various cytokines and numerous growth factors such as NGF and artemin that are upregulated in the skin following inflammation [5]. Behavioral studies of TRPV1-/- mice revealed that they exhibited mild attenuation of their responses to noxious thermal stimuli. However, the mice strikingly did not develop enhanced sensitivity to heat following inflammation, suggesting that TRPV1 is essential for the development of inflammation-induced heat hyperalgesia. In contrast, the TRPV1-/- mice did develop mechanical hyperalgesia following inflammation [6,7].

TRPV1 is contained in a subpopulation of small and medium size sensory neurons [1,8]. In mice, TRPV1 is found in a higher proportion of muscle and visceral sensory neurons than cutaneous ones [9]. In uninjured murine fibers innervating hairy skin, TRPV1 is localized specifically in a subpopulation of C-fiber nociceptors that respond to heat (CH-fibers) but not mechanical or cold stimuli [10,11]. The majority of C-fibers innervating the skin are C-polymodal (CPM) afferents responding to both mechanical and thermal stimuli, the vast majority of which have been shown to be non-peptidergic IB4 positive fibers that lack TRPV1 [10,12]. Notably, in TRPV1-/- mice these CPM fibers exhibit normal heat responses [12] although they lack functional CH fibers [10].

The fact that TRPPV1-/- mice do not develop heat hyperalgesia following inflammation suggests the possibility that the remaining functional heat sensitive CPM fibers may not be sensitized to heat following inflammation. Whereas many studies have demonstrated heat sensitization of nociceptors following inflammation [13-15] others have reported conflicting results [16-18]. Based on these findings we hypothesized that inflammation-induced thermal hyperalgesia is normally mediated primarily by sensitization of TRPV1-immunoreactive CH-fibers. Conversely, we predicted that the heat sensitivity of CPM-fibers that lack TRPV1 should remain relatively unchanged, but that they should display an increased sensitivity to mechanical stimuli, driving inflammation-induced mechanical hyperalgesia. Here we directly address this hypotheses by first examining the affect of CFA-induced inflammation on the response of cutaneous C-fibers to skin stimulation in naïve C3H/Bl6 mice using an *ex vivo *somatosensory system preparation. Next we repeated these studies in both TRPV1-/- mice combing both behavioral and electrophysiological analyses to assess the effects of inflammation on nociceptor function.

## Methods

### Animals

All animals were 4-6 weeks old and obtained through breeding at the animal facility of the University of Pittsburgh. Mice were housed in group cages, maintained on a 12:12 light-dark cycle in a temperature controlled environment (20.5°C) and given food and water ad libitum. All studies were carried out in accordance with the guidelines of the University of Pittsburgh Institutional Animal Care and Use Committee and the National Institute of Health Guide for the Care and use of Laboratory Animals.

### Behavioral testing of thermal sensitivity and the induction of inflammation

Thermal sensitivity of all animals was examined using the Hargreaves test [19]. Animals were placed into individual chambers on a glass plate maintained at 30°C and allowed to acclimate for 90 min (for details see Malin et al., 2006) [5]. A radiant heat stimulus (Hargreaves apparatus set to 15% intensity; IITC, Woodland Hills, CA) was applied to the plantar surface of the foot and the response latencies to noxious thermal stimulation were measured before and after inflammation.

Inflammation was induced in all animals by lightly anaesthetizing them using isoflurane prior to injecting subcutaneously 20 μl of 1:1 emulsion of complete Freud's adjuvant containing 1 mg/ml *Myobacterium tuberculosis *(CFA; Sigma) and 0.9% NaCl into the dorsum of the foot [20]. In a first series C3H/Bl6 mice were sacrificed 1, 3, or 5 days after CFA injection. The second set of experiments was conducted using TRPV1-/- and C57/Bl6 control animals which were sacrificed 3 days after the injection. In order to measure the degree of inflammation, the width and height of each injected foot was measured at the midpoint of the hindpaw (half way between the toes and heel) using calipers. All animals were then analyzed using the ex vivo preparation. In all the different stages (e.g. behavior, CFA injection and physiological recordings) the experimenters were blinded to the genotype of the mice.

### Ex vivo preparation

The *ex vivo *preparation was used as previously described by McIlwrath et al. [21]. In short, the animal was anaesthetized using a mixture of ketamine (90 mg/kg) and xylazine (10 mg/kg) and then transcardially perfused with chilled (6°C) oxygenated (95% O_2_-5%CO_2_) aCSF in which Na^+ ^was replaced with sucrose (sucrose aCSF, in mM: 254 sucrose, 1.9 KCl, 1.2 KH_2_PO_4_, 1.3 MgSO_4_, 2.4 CaCl_2_, 26.0 NaHCO_3_, 10.0 D-glucose). The skin from the right hindlimb, saphenous nerve, lumbar DRG, and the spinal cord were excised in continuum and transferred to the recording chamber filled with normal aCSF in which the sucrose was replaced with 127 mN NaCl. Depending on the experiment, encapsulated CFA associated with the skin was removed using a blunt syringe to prevent its spread in the bath. The skin was pinned epidermal side up on a rigid platform and the bath temperature maintained at 31°C.

Sensory neuron somata were impaled using quartz microelectrodes (> 100 MΩ) filled with 5% neurobiotin (Vector Laboratories, Burlington, CA) in 1 M potassium acetate. Mechanical (paint brush) and thermal (cold (0-2°C) and hot saline (52-54°C)) stimuli were used to identify cutaneous receptive fields (RF). All electrophysiological data was digitized using a Power 1401 data acquisition unit and Spike 4.0 software (Cambridge Electrical Design Ltd., Cambridge, UK) and analyzed offline. The conduction velocity was calculated from the distance between the stimulating electrode on the saphenous nerve and recording microelectrode in the DRG and the spike latency. Mechanically sensitive RFs were characterized using a computer-controlled mechanical stimulator (Aurora Scientific Inc., Ontario, Canada) with a circular diameter of 1 mm. An ascending series of constant force stimuli from 1 - 100 mN each lasting 5 sec were applied to the RF with 30 sec resting intervals between stimuli. The RF was then cooled to ~ 6°C and after the temperature reached bath level again, heated to 52°C (15 sec ramp) using a computer controlled peltier device with a 5 mm^2 ^contact area. For fibers that did not respond to mechanical stimulation thermal RFs were located using syringes containing hot (52°C) and cold (0°C) saline and were characterized with thermal stimuli. It should be pointed out that once we had located the cell's RF in these cases we reapplied mechanical stimuli (glass rod or >2 g von Frey hair) to confirm the lack of mechanical sensitivity. We considered any response to mechanical stimulation regardless of the intensity of the stimulus to show mechanical sensitivity. Finally, the cell was iontophoretically injected with neurobiotin. At the end of the experiment, DRGs containing labeled cells were removed and immersion fixed using 4% paraformaldehyde in 0.1 M phosphate buffer (PB) for 30 min.

### Analysis of electrophysiological data

Except for the heat ramp, all data is presented as the mean ± standard error of the mean (SEM). Their statistical difference was tested using Student's t-tests. Only the responses of C-fibers to the heat ramp were normalized to reflect the information that is transmitted to the neuronal circuits in the spinal cord. Normalization was achieved by multiplying the mean spikes per degree Celsius with the ratio of afferents responding at a given temperature and the total number of cells recorded. Statistical significance of the responses to the ascending mechanical stimuli was determined by using the Students' t-test and to the heat ramp by performing 2-way ANOVAs with a Bonferoni posthoc test.

### Immunohistochemistry

Dorsal root ganglia were individually embedded in gelatin, postfixed in 4% paraformaldehyde, cryprotected in 20% sucrose, and sectioned at 60 μm. Frozen sections were collected in PB and reacted over night with rabbit anti-TRPV1 (Oncogene Research, San Diego, CA (now CalBiochem)) antibody and IB4 conjugated with Cy3 (Molecular Probes, Eugene, OR, USA). The neurobiotin-filled cell was visualized utilizing avidin-FITC (Vector Laboratories, Burlingame, CA) and antibody staining using a secondary antibody made in donkey conjugated to Cy5 (Jackson ImmunoResearch Laboratories Inc., West Grove, PA). Finally tissue was analyzed using an Olympus FluoViewTM 500 laser scanning confocal microscope (Olympus America Inc., Melville, NY).

The number of TRPV1-positive cells was analyzed in four L3 DRGs from naive mice and three L3 DRGs from each nerve injured group. DRGs were taken after electrophysiological experiments and stained as described above. Tissue sections were analyzed as reported previously [9]. In brief, three nonconsecutive sections were randomly chosen, and 15 μm stacks with 3-μm-thick optical sections were captured using a 40× oil-immersion objective. Multiple optical stacks were taken of each selected tissue section, and visual confirmation was used to avoid analyzing cells twice. The number of TRPV1-positive cells counted and averaged in the top and bottom optical section of each stack. The total number of labeled cells per section was determined and reported as mean ± SEM.

## Results

### Effects of inflammation on cutaneous nociceptor response properties

In the first series of experiments, we used an *ex vivo *skin/saphenous nerve/DRG/spinal cord preparation to characterize the peripheral response properties of different functional types of cutaneous sensory neurons in naïve and CFA injected C3H/Bl6 mice. In this set of experiments, a total of 270 characterized cutaneous A- and C-fiber nociceptors were recorded in naïve (14 male and 12 female; n = 142) and inflamed (7 male and 5 female; n = 128) C3H/Bl6 mice. Nociceptors were identified based on their peripheral response properties and the presence of an inflected somal action potential [22]. Afferents conducting faster than 1.2 m/s were classified as A-fibers while all others were classified as C-fibers. A-fiber nociceptors were divided into two groups; those that responded to both mechanical and thermal stimuli (APM) and those that only responded to mechanical (AM). C-fibers were further divided based on their peripheral response properties to mechanical and thermal stimuli [10]. These groups included: fibers that responded to mechanical and heat and sometimes cold stimuli (CPM); C-mechanocold (CMC) fibers responded to mechanical and cold stimuli on the cutaneous receptive field (RF); C-mechano (CM) fibers responded to mechanical but not to thermal stimulation of the skin (hot or cold); C-cold (CC) fibers responded only to cold/cooling and not to mechanical or heat stimuli; C-heat (CH) fibers responded to heat, but not to mechanical or cold stimuli. There were no differences found between the response properties of cutaneous sensory neurons recorded in naïve male and female mice (e.g. CPM heat thresholds male = 42.1 ± 0.6°C; female 40.8 ± 1.0°C; p = 0.25). There were also no differences between fibers recorded in male and female mice following CFA injection (e.g. CPM heat thresholds; male 38.8 ± 1.1°C; female 38.6 ± 1.0°C; p = 0.9). Therefore the results of male and female mice were combined for analysis.

**Figure 1:**
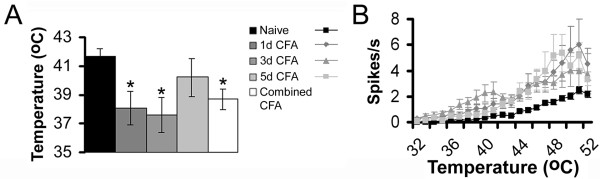
**Inflammation-induced heat sensitization of TRPV1-negative CPM-fibers**. **A) **Heat thresholds of CPM-fibers were significantly reduced 1 (dark grey bar) and 3 days (medium grey bar) after the injection of CFA and indistinguishable from those in naïve animals (black bar) at day 5 (light grey bar). The combined heat threshold of all time points after inflammation (white bar) was significantly lower than in naïve animals. **B) **The firing rates of CPM-fibers to noxious heat stimuli (grey symbols) were significantly higher at all time points after inflammation when compared to those from naïve mice (black squares).

**Figure 2:**
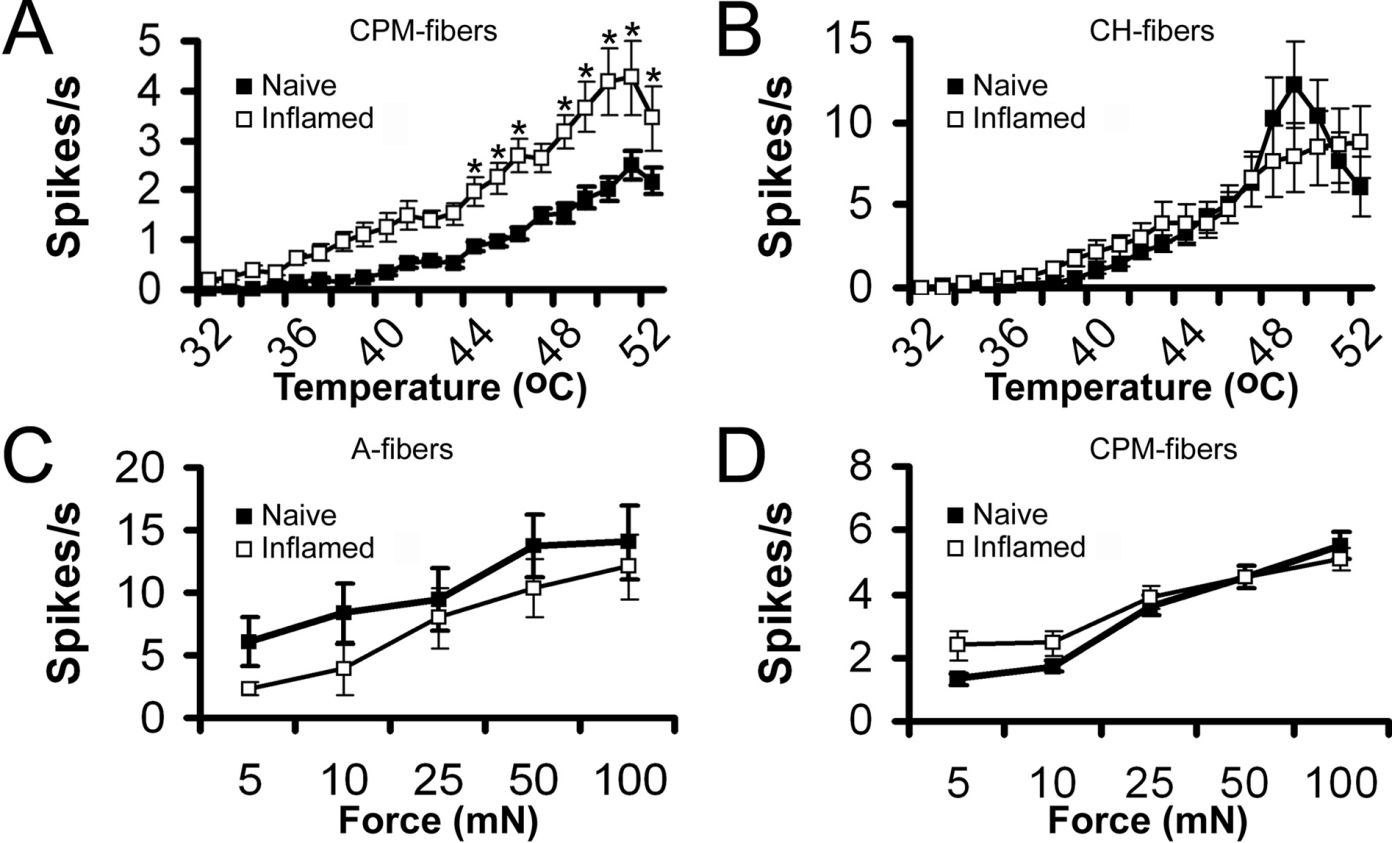
**CH-fibers lack heat sensitization and AM and CPM-fibers lack mechanical sensitization after inflammation**. **A) **After inflammation, CPM-fibers encode heating of the cutaneous receptive field with significantly higher firing rates while **B) **CH-fibers are not different. Neither A-fibers in **C) **or CPM-fibers in **D) **responded with increased firing rates to ascending series of constant force stimuli after inflammation.

In naïve C3H/Bl6 mice the average heat threshold of CPM-fibers was 41.7 ± 0.5°C, n = 73; (Fig. 1A). One and three days after subcutaneous injection of CFA into the dorsum of the hindpaw, the average heat threshold of CPM-fibers was significantly reduced to 38.1 ± 0.8°C, at day 1 (p < 0.001) and to 37.6 ± 1.3°C at day 3 (p < 0.001). By day 5, the heat threshold of CPM-fibers had returned to near naïve levels (40.2 ± 0.7°C). When combining CPM-fibers from all 3 time points after inflammation, the resulting average heat threshold was still significantly lower than those in naive animals (CPM = 38.7 ± 0.8°C, n = 46; p < 0.001). The magnitude of the responses of CPM-fibers in naïve and inflamed animals also differed. One day after inflammation, CPM-fibers responded to the heat ramp with significantly higher mean firing rates at 38°C and higher temperatures (2-way ANOVA, Bonferoni p < 0.001; Fig. 1B). This persisted at 3 days post CFA injection when mean firing rates were significantly higher at temperatures above 42°C (2-way ANOVA, Bonferoni p < 0.05; Fig. 1B ) and at 5 days post CFA injection at temperatures higher than 44°C (2-way ANOVA, Boneroni p < 0.05; Fig. 1B ). As with heat thresholds, the combined responses from all three time points was also significantly higher to noxious temperatures above 44°C (2-way ANOVA, Bonferoni p < 0.001; Fig. 2A). However, there was no change in the response of CPMs to cold, or to mechanical stimulation (e.g. naïve CPM mechanical threshold 14.8 ± 2.0 mN vs inflamed 18.2 ± 2.9 mN). This was also true for the other C-fiber types (CMC, CM, CC). Whereas there was a trend toward a decrease in the mean heat threshold of CH fibers, this change was not statistically significant (naïve = 41.8 ± 1.6°C, n = 11; combined inflamed = 39.1 ± 1.7°C, n = 11; p = 0.3). The mean firing rate of the CH neurons during application of the heat ramp after inflammation was also unchanged (Fig. 2B). Following inflammation we also recorded from a total 14 mechanically sensitive A-fibers (AM); none of these AM nociceptors responded to heat. As we found for CPM fibers (Fig. 2D), the response to mechanical stimulation was unchanged in AM nociceptors following inflammation (Fig. 2C).

### The role of TRPV1 in inflammation

The results of these initial experiments suggested that the CPM fibers (that do not normally express TRPV1) are sensitized to heat following inflammation. In order to determine whether this inflammation-induced sensitization was truly a TRPV1-independent process, we next examined the effects of inflammation on the response properties of cutaneous CPM fibers in TRPV1-/- and wildtype mice. In this series of experiments we focused on a single time point (3 days) following CFA injection.

**Figure 3:**
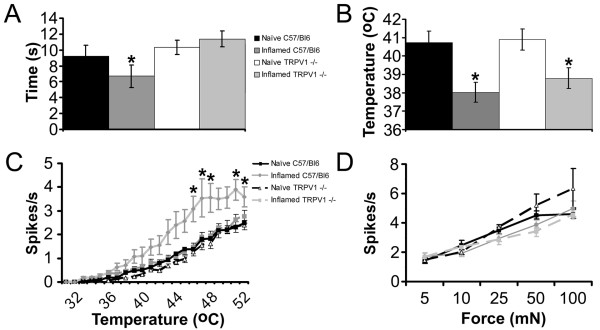
**CPM-fibers exhibit decreased heat thresholds in inflamed TRPV-/- mice**. **A) **Only C57/BL6 mice have reduced latencies when responding to a radiant heat stimulus to the plantar surface of the hindpaw after inflammation. As previously described, in TRPV1-/- mice, the response latency to this stimulus is not different after inflammation. **B) **CPM-fibers that do not stain for TRPV1 have reduced heat thresholds after inflammation in TRPV1-/- mice. **C) **Unexpectedly, the firing rates of CPM-fibers to stimulation with a heat ramp are only significantly increased after inflammation in C57/BL6 animals. **D) **The mechanical response properties of CPM-fibers were not different after inflammation in both C57/BL6 and TRPV1-/- mice.

Inflammation was induced as before by a subcutaneous injection of CFA into the dorsum of the foot of adult male TRPV1-/- (n = 10) and wildtype (WT) C57BL6 (n = 10) mice. Three days later, all mice exhibited inflamed hindpaws. Behavioral testing revealed that WT C57BL6 mice had significantly shorter paw withdrawal latencies in response to noxious radiant heat delivered to the plantar surface of the inflamed hindpaw (naïve C57BL6 = 9.2 ± 1.4 s, inflamed C57BL6 = 6.7 ± 1.4 s, p < 0.05; Fig. 3A), while TRPV1-/- mice did not develop thermal hyperalgesia (naïve TRPV1-/- = 10.3 ± 0.9 s, inflamed TRV1-/- = 11.4 ± 1.0 s).

Following behavioral testing we used the *ex vivo *preparation to determine if CPM neurons in these same TRPV1-/- and C57/Bl6 mice exhibited sensitization as seen in the C3H/Bl6 mouse strain. We intracellularly recorded from a total of 307 cutaneous C-fibers in naïve and inflamed C57BL6 and TRPV1-/- animals. Similar to that observed for CPM-fibers in C3H/Bl6 mice, the average heat threshold of CPMs in C57BL6 wildtype mice dropped significantly from 40.8 ± 0.6°C (n = 51) in naïve mice to 38.0 ± 0.5°C (n = 54; p < 0.01) 3 days after CFA injection (Fig. 3B). In addition, these fibers exhibited an increased sensitivity to heat evidenced by an increased firing rate in response to noxious heat temperatures (2-way ANOVA, Bonferoni p < 0.001) (Fig. 3C). In TRPV1-/- mice, the average heat threshold of CPM-fibers was also significantly reduced 3 days after inflammation (naïve TRPV1-/- = 40.9 ± 0.6°C, n = 62; inflamed TRPV1-/- = 38.7 ± 0.6°C, n = 45; p < 0.01). However, unlike wildtype mice, CPM-fibers from inflamed TRPV1-/- animals did not exhibited any change in the mean firing rates during the heat ramp (Fig. 3C).

The response of CPM-fibers to an ascending series of constant force stimuli was also not different after inflammation in WT and TRPV1-/- mice (Fig. 3D). Interestingly, the lack of increased mechanical and thermal sensitivity of CPM fibers in inflamed TRPV1-/- mice was correlated with a trend towards reduced paw edema following CFA injection in these animals. The average area of the inflamed foot 16.2 ± 1.2 mm^2 ^in C57BL6 and 12.9 ± 1.1 mm^2 ^in TRPV1-/- mice (p = 0.06). Additionally, in agreement with the previous set of experiments, CH fibers in wildtype mice were unaffected by inflammation as their heat thresholds (naïve, 40.5 ± 2.4°C, n = 7; inflamed, 39.9 ± 1.9°C, n = 8) and firing rates were also unchanged (data not shown).

**Figure 4:**
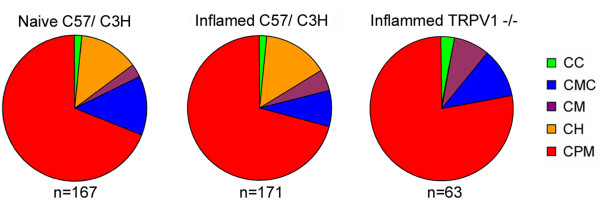
**Distribution of C-fiber types is unchanged following inflammation**. The prevalence of CH-fibers does not increase after inflammation compared to naïve mice. (p value < 0.17; χ^2 ^test.) Neither naïve (not shown) nor inflamed TRPV1-/- animals have CH-fibers.

In addition to examining the peripheral response properties of the cutaneous nociceptors, we also compared the distribution of the different types of unmyelinated cutaneous nociceptors before and after inflammation in wildtype C57/Bl6, C3H/Bl6 and TRPV1 KO mice (Fig. 4). There were no differences in distribution of C-fibers between wildtype C3H/Bl6 and C57/Bl6 mice before or after inflammation; therefore, the data was combined for presentation. The largest group of identified C-fibers in both naïve and inflamed animals was the CPM-fibers (naïve = 68.9%; combined inflamed = 70.8%). In naïve mice, we observed similar numbers of CH- (13.2%), CMC (13.2%), CMs (3%) and CC (1.8%) fibers. After inflammation there was not a significant change in the distribution of fiber types (χ^2^, p < .17). We have previously reported a lack of CH-fibers in naïve TRPV1-/- animals [10]. Here we also found that inflamed TRPV1-/- mice lack CH fibers (not shown). In naïve TRPV1-/- 75.3% of all identified C-fibers were CPMs, 9.4% were CMs, 4.7% were CCs and the remaining 10.6% were CMCs. The distribution of identified C-fibers in TRPV1-/- mice was similar after inflammation; 77.8% were CPMs, 7.9% were CMs, 3.2% were CC, and 11.1% were CMC-fibers.

### Immunohistochemistry

**Figure 5:**
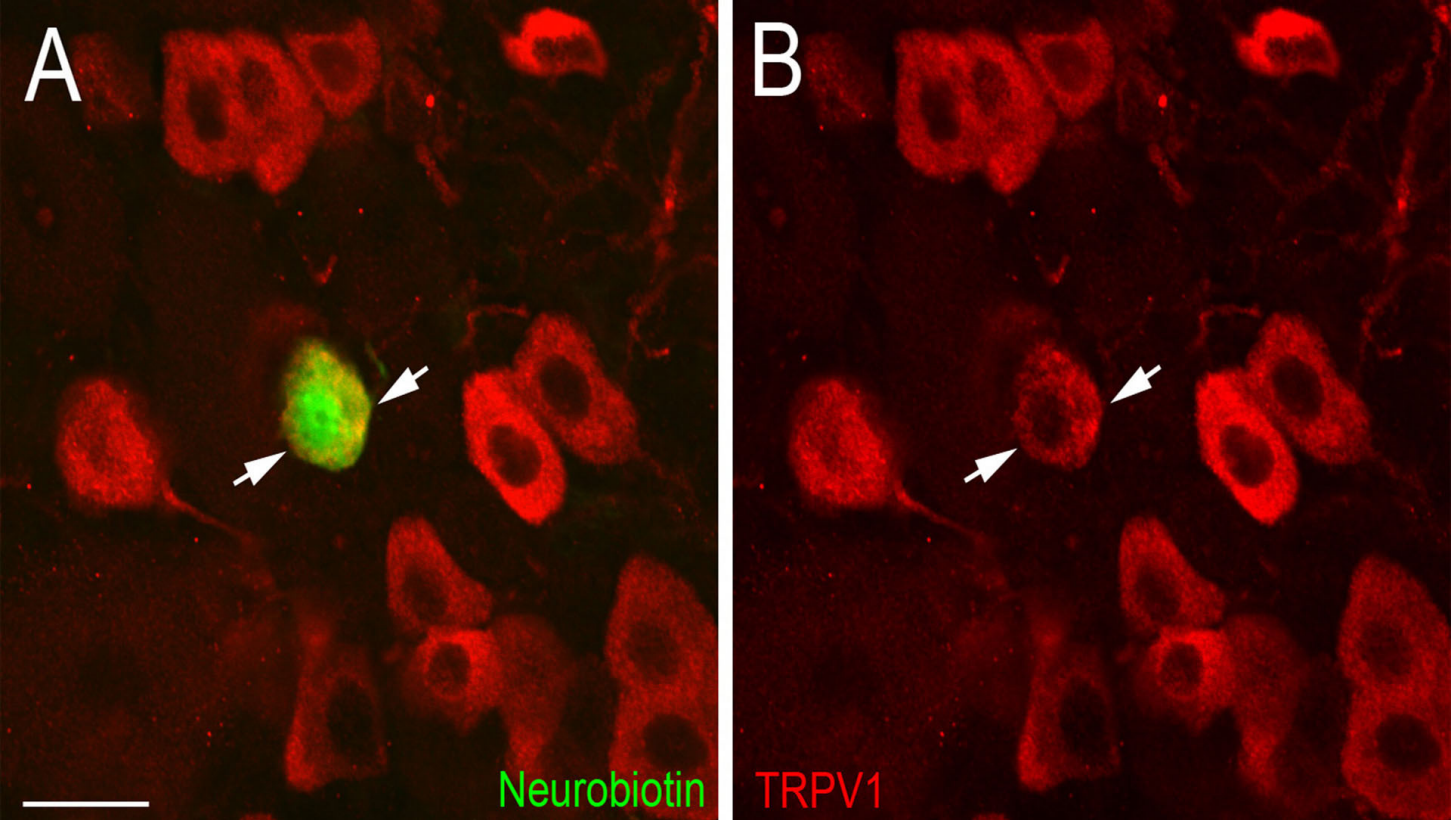
**Example of TRPV1-positive CPM-fiber following inflammation**. Light micrographs of a L3 DRG from the *ex vivo *recording preparation containing a neurobiotin-filled polymodal C-fiber (CPM) immunoreactive for TRPV1. **A) **A Neurobiotin labeled (green) CPM neuron (arrows) recovered from the *ex vivo *preparation overlaps with TRPV1 immunoreactivity (red). **B) **Immunostaining for TRPV1 (red) shows that the same neurobiotin-filled CPM neuron in **A **is immunopositive for TRPV1 (arrows). Scale bar, 20 μm.

After the characterization of the cutaneous RFs, selected neurons were filled with neurobiotin and the DRGs later stained for IB4 binding and TRPV1 immunoreactivity. In Table 1, 81% (31/38) of characterized CPM-fibers in naïve mice labeled for IB4 and none (0/34) were immunoreactive for TRPV1. After inflammation, a similar number of CPM-fibers 83% (30/36) bound IB4. However, following inflammation 11% of CPMs (4/36) were found to stain positively for TRPV1 (Fig. 5) and these cells did not bind IB4 suggesting that some of the CH cells may have acquired mechanical sensitivity following inflammation. In addition, all 10 of the CH fibers from both naïve and inflamed mice were found to be TRPV1-positive and IB4-negative. Cell counts in the same ganglia also revealed that the numbers of TRPV1 positive sensory neurons in the L3 ganglia C57/Bl6 mice were unchanged following CFA injections (naive 66.6 ± 4.0, *vs *inflamed 72.9 ± 4.4, p = 0.29; t-test).

**Table 1:** TRPV1 and IB4 staining of identified CPM-fibers in naïve and inflamed mice

CPM fibers	IB4-positive ratio	TRPV1-positive ratio
Combined naïve C3H/BL6 and C57/BL6	31/38	0/34

Combined inflamed C3H/BL6 and C57/BL6	30/36	4/36

Identified C-polymodal fibers in naïve and inflamed wildtype animals for IB4 binding and TRPV1 immunoreactivity. Only after inflammation are TRPV1 positive CPM-fibers observed.

Another difference between naïve and inflamed WT CPMs was a significant decrease in mean conduction velocity following inflammation (naïve = 0.53 +/- 0.01 m/s *vs *inflamed = 0.5 +/- 0.01 m/s; p value < 0.05; student's t-test). Interestingly, this change in CPM conduction velocity was not observed in the TRPV-/- mice (naïve = 0.53 +/- 0.01 m/s *vs *inflamed = 0.51 +/- 0.01 m/s; p value >0.05; student's t-test) mice after inflammation. This drop in conduction velocity was also not observed for any other fiber type. For example, the mean conduction velocity of CH fibers was unchanged following inflammation (naïve = 0.36 +/-0.02 m/s *vs *inflamed = 0.36 +/- 0.01 m/s).

## Discussion

Based on previous studies from our lab and others, we hypothesized that mechanically insensitive TRPV1-positive CH fibers would be sensitized to heat, while CPM fibers, that normally do not express TRPV1, would not be sensitized in inflamed mice. However, following inflammation we found no change in the heat sensitivity of CH fibers while CPM fibers exhibited reduced heat thresholds and increased firing rates during heating of the skin in both C3H/Bl6 and C57Bl6 wildtype, but only exhibited a decease in heat threshold in TRPV1-/- mice. We also found, contrary to expectations, that mechanical sensitivity was unchanged in this population of fibers in all mouse strains (wildtype and TRPV1-/-) following inflammation.

### Lack of change in mechanical sensitivity following inflammation

We did not observe any change in the mechanical sensitivity of either A- or C-fibers following CFA injection into the dorsum of the hindpaw in these preparations, consistent with previous studies in rodents showing a lack of mechanical sensitization of C-fibers following inflammation [14,15,23]. However, this differs from other studies showing increased mechanical sensitivity after peripheral inflammation [16,24]. One possible reason for this discrepancy is the nature of the *ex vivo *preparation. Previous studies using intact preparations have shown that during inflammation, edema produces increased tension of the skin and that increased tension of the skin is correlated with a reduction in mechanical thresholds of afferent fibers [25,26]. In the *ex vivo *preparation, the skin is freed from the underlying tissue, removing any stretching caused by the edema. Moreover, those reporting mechanical sensitization used mostly *in vivo *preparations [16], while those that reported a lack of sensitization generally have used some type of *ex vivo *preparation [15,23]. Thus, the results observed here suggest that increased mechanical sensitivity of nociceptors following inflammation requires changes in tensile loading of the skin and is not solely due to changes in receptor/channel function by inflammatory mediators. However, it should be noted that some *in vivo *studies have demonstrated mechanical sensitization within short time frames following injection of inflammatory mediators before edema formation suggesting that changes in receptor/channel function may also play a significant role in mechanical sensitization [e.g. [24], but also see, [27,28]].

### Changes in CH-fibers following inflammation

Mechanically insensitive C-fibers (MIA) have been described in several species, including mouse [10,12,29], rat [30] non-human primates [24,31], and humans [32,33]. Similar to our recent findings in mice [10], many MIA afferents in humans and other species can be activated by heat stimuli and capsaicin [32].

The results from previous studies [6,7,10,12,34] suggest that the population of TRPV1 positive, mechanically insensitive CH fibers play an important role in the development of inflammation induced heat hyperalgesia. Here we found an apparent lack of sensitization of these fibers following inflammation. Although the total number of CH fibers examined in each case is relatively small (11) when compared to the number of CPM fibers (46), this observation is not consistent with the idea that CH fibers play an important role. There are a couple of possible explanations for these findings. It is possible that the process of establishing the *ex vivo *preparation normally sensitizes these specific afferents thus masking any inflammation induced sensitization. While we do not know of any evidence for this possibility, it is something that we cannot completely rule out. Another possible explanation is recruitment of previously silent CH fibers. Previously, we found that there was an apparent increase in the numbers of functional CH fibers following saphenous nerve regeneration and that some CH fibers gained mechanical sensitivity after nerve injury [35]. While we did not observe the same significant increase in functional CHs here, we have found that some CH fibers are apparently gaining mechanical sensitivity as indicated by the number of the CPM fibers (11%) stained positively for TRPV1 (in naïve animals this number is 0 [10,35]). We and others have reported increases in expression and/or protein levels of TRPV1 following peripheral injury [35-40]. While there was no change in the numbers of TRPV1 positive cells in the DRG observed in the current study, two of the previous studies report an increase in the numbers of TRPV1 positive cells following inflammation. A major difference between those studies and the current one is that they were carried out in rat, where the distribution of TRPV1 is quite different than in mouse. For example, there is a large degree of overlap between TRPV1 and IB4 binding in rat and very little overlap seen in mouse [12]. The current finding is also in agreement with our previous observations in mice following nerve injury [35] where we found an increase in TRPV1 expression but no increase in the number of TRPV1 positive cells. In addition, in a separate study in our laboratories no change in the number of TRPV1-positive cells following inflammation was observed (B.M. Davis, unpublished observation). Therefore the difference observed in the current studies and the previous ones could be due to species differences. However, it should also be noted that since we are counting the total numbers of cells, small changes in the numbers of fibers innervating the inflamed skin maybe masked.

We have also previously shown that CH fibers have significantly slower CVs than CPM fibers [10] and here, as in the previous study following regeneration [35], we found a significant decrease in the average CV of CPMs after inflammation. Together these findings suggest that some of the mechanically insensitive CHs have changed phenotypes by acquiring mechanical sensitivity. This change in phenotype should result in a decrease in percentage of C-fibers innervating inflamed skin characterized as CH fibers. However, this was not observed suggesting the possibility that some previously "silent" (mechanical and heat insensitive) fibers gained heat sensitivity following inflammation. However we cannot completely rule out other possible explanations for these findings. For example, there maybe a population of silent TRPV1-positive CPM fibers that have slower CV than naïve CPM fibers, and that these fibers gain mechanical and heat sensitivity following inflammation.

It has also been shown that mechanically insensitive cutaneous fibers can rapidly gain mechanical sensitivity following injection of inflammatory mediators (e.g. Davis et al., 1993) [24]. Additionally, microneurography studies in humans showing that following capsaicin application, MIA-fibers can quickly become mechanically sensitive [32] and investigators report that similar changes in this set of fibers are found in patients with chronic pain disorders [41,42]. Taken together with the results presented here, these findings suggest that it is the normally mechanically insensitive CH-fibers that exhibit pronounced plasticity following peripheral injury and could contribute to both heat and mechanical hyperalgesia observed following inflammation.

### TRPV1-independent heat sensitization of CPM fibers

In the present study we found that inflammation induced sensitization of TRPV1-negative CPM-fibers to heat in two different strains of wildtype mice and this change was correlated with heat hyperalgesia in the C57/Bl6 mice. The results in wildtype mice strongly suggested that this was a TRPV1-independent process, and additional experiments carried out in the inflamed TRPV1-/- mice confirmed that at least part of the heat sensitization process of CPM-fibers (the decrease in heat threshold) is independent of TRPV1. One possible mechanism for these changes could be changes in the expression of purinergic receptors following inflammation. It has been shown that this population of IB4-binding fibers also contains both P2X3 or P2Y1 receptors [43,44] and it has also been shown that they can play a role in changes in neuronal sensitivity [45-47]. In addition, we have recently shown that this population of cutaneous CPM fibers undergo a very similar decrease in heat thresholds following peripheral nerve injury and regeneration, and that this decrease was correlated with changes in the expression of these two purinergic receptors P2X3 and P2Y1 [35].

In this study, we have also confirmed the earlier reports [6,7] showing that TRPV1-/- mice do not develop heat hyperalgesia following inflammation. These findings suggest that although CPMs show a significant decrease in heat thresholds, this change, in and of itself, is not sufficient to induce behavioral changes. It is quite possible that the full sensitization of CPM fibers, including increased firing rates, is necessary to drive behavioral changes. However, It is important to note that Cavanaugh et al., [34] have reported recently that the population of IB4-positive/TRPV1-negative CPM fibers expressing the G protein coupled receptor Mrgprd, can be ablated without diminishing acute thermal pain in mice. The results presented here are consistent with these findings and further suggests the possibility that these fibers cannot by themselves signal enhanced acute heat pain following inflammation. However, it should also be noted that TRPV1-/- mice have relatively normal acute thermal pain detection [6,7] indicating that mechanisms exist that allow mice lacking TRPV1-positive CH fibers, or for that matter any fiber containing TRPV1, to detect noxious thermal stimuli.

### A possible effecter role for fibers containing TRPV1 in inflammation-induced heat hyperalgesia

Here we have confirmed our earlier findings that TRPV1-/- mice lack CH-fibers [10] and show that they are still absent following inflammation. However, TRPV1-/- mice have been shown to develop heat hyperalgesia following neuropathic injury [6], suggesting the possibility that TRPV1 has a significant contribution to the inflammatory response that is not essential for increased heat sensitivity after neuropathic injuries. While it is possible that this difference reflects compensatory mechanisms in the constitutive knock-out mice, support for the former possibility can be found in recent reports showing that TRPV1-/- mice exhibit less edema and swelling following CFA-induced inflammation [48,49] but also see Davis et al., 2000 [7]. Given the propensity for TRPV1 to be sensitized by endogenous factors and that activation of TRPV1 has been shown to evoke release of vasoactive peptides in the skin (e.g CGRP) [50,51] these fibers may be playing an important role in the neurogenic inflammatory response. For example, it has been shown that TRPV1 sensitivity can be significantly modulated by many of the components of the inflammatory milieu (e.g. bradykinin, and NGF) present in inflamed skin as well as by local decreases in pH [2,52-54], that in turn could result in local peptide release in the inflamed tissue contributing to neurogenic inflammation [3]. It is also of interest to note that in an earlier study in the pig, Lynn et al., (1996) [55] reported that a vasodilatory response was elicited by stimulation of CH-fibers, but not CPM-fibers.

One possible mechanism that could be responsible for the increase in the magnitude of the response to heat in IB4-positive CPM fibers is modulation of the M-current. The M-current has been shown to be present in CPM fibers innervating mouse skin that also contain the mas gene related G-protein coupled receptor D [56]. Peptides can inhibit the M-current either directly [57] or indirectly by inducing the release of inflammatory mediators such as bradykinin [58]. Thus, the increased responsiveness to heat in the CPM population of fibers could be mediated by peptide release from TRPV1-positive CH fibers.

## Conclusion

The data presented here indicate that there are two types of C-fibers encoding heat stimuli that have very different roles. Although CPM-fibers lacking TRPV1 constitute the majority of C-fibers innervating the skin, decreased heat thresholds in these fibers alone appears to be insufficient for establishment of thermal hyperalgesia after inflammation. In contrast, TRPV1-positive mechanically insensitive CH-fibers appear to play an essential role in the establishment of thermal hyperalgesia after inflammation. It will be important in the future to determine the role of CPM neuron sensitization after peripheral inflammation to fully understand their function.

## Competing interests

The authors declare that they have no competing interests.

## Authors' contributions

HRK designed and help conduct experiments and wrote manuscript, SLM conducted experiments and help write early drafts of manuscript, JJL conducted experiments and initial data analysis, SAM conducted behavioral experiments, CEA conducted cells counts and helped with data analysis, MPJ data analysis and contributed to writing manuscript, BMD helped with experimental design and behavioral experiments and contributed to writing of the manuscript.

All authors have read and approved the final manuscript.
